# ISLRWR: A network diffusion algorithm for drug–target interactions prediction

**DOI:** 10.1371/journal.pone.0302281

**Published:** 2025-01-30

**Authors:** Lu Sun, Zhixiang Yin, Lin Lu

**Affiliations:** 1 School of Mathematics, Physics and Statistics, Institute for Frontier Medical Technology, Center of Intelligent Computing and Applied Statistics, Shanghai University of Engineering Science, Shanghai, China; 2 Shanghai Xinhao Information Technology Co., Ltd., Shanghai, China; Chinese Academy of Sciences, CHINA

## Abstract

Machine learning techniques and computer-aided methods are now widely used in the pre-discovery tasks of drug discovery, effectively improving the efficiency of drug development and reducing the workload and cost. In this study, we used multi-source heterogeneous network information to build a network model, learn the network topology through multiple network diffusion algorithms, and obtain compressed low-dimensional feature vectors for predicting drug–target interactions (DTIs). We applied the metropolis–hasting random walk (MHRW) algorithm to improve the performance of the random walk with restart (RWR) algorithm, forming the basis by which the self-loop probability of the current node is removed. Additionally, the propagation efficiency of the MHRW was improved using the improved metropolis–hasting random walk (IMRWR) algorithm, facilitating network deep sampling. Finally, we proposed a correction of the transfer probability of the entire network after increasing the self-loop rate of isolated nodes to form the ISLRWR algorithm. Notably, the ISLRWR algorithm improved the area under the receiver operating characteristic curve (AUROC) by 7.53 and 5.72%, and the area under the precision-recall curve (AUPRC) by 5.95 and 4.19% compared to the RWR and MHRW algorithms, respectively, in predicting DTIs performance. Moreover, after excluding the interference of homologous proteins (popular drugs or targets may lead to inflated prediction results), the ISLRWR algorithm still showed a significant performance improvement.

## Introduction

Drug–target interactions (DTIs) prediction is essential for discovering new drugs and potential targets. Traditional methods for developing new drugs require several chemical and clinical trials [1], with a long R&D cycle and massive capital consumption. The process from lead identification to clinical trials takes at least 12 years, with an R&D cost of up to 1–1.8 billion USD [2, 3]. Therefore, many scholars have proposed using machine learning and computer-aided tools to replace the traditional pre-discovery work of drug R&D to improve efficiency and reduce costs [4–6].

Computer technologies such as machine learning are widely used in various aspects of drug screening [7, 8], instead of the need to perform many repetitive tasks [9], and provide direction and range for aimless drug experiments. The DTIs prediction task is a critical and limiting step for discovering potential targets and new drugs [10, 11]. In addition, drug–drug interactions (DDIs) [12], protein–protein interactions (PPIs) [13], miRNA–disease correlation prediction [14, 15], and drug–target binding affinity (DTA) prediction [16] are typical computer-aided drug screening tasks. DDIs are concerned with drug side effects and toxicity, where patients may experience life-threatening pharmacological toxicity or side effects with severe clinical consequences when taking multiple drugs [17, 18]. PPIs focus on the physiological functions of proteins and assist with understanding the interactions between cellular molecules and viral microorganisms [19]. The study of miRNA–disease interrelationships is essential to understanding human disease pathogenesis because abnormal expression and regulation of some miRNAs can lead to the development of certain diseases [15, 20]. DTA prediction focuses on successive values of the strength of the interaction of a drug with a protein or a gene locus [21].

This study focuses on DTIs prediction given that it represents a more direct means of learning potential drug–target pairs [10, 11]. DDIs focus on drug characteristics, such as drug toxicity and side effects, which are directly significant in drug use and clinical application [12, 17, 18]. DDIs predictions are usually based on semantic relationships, such as knowledge graphs, in which the side effects, dosages, and indications of a drug are deposited into a certain network node. The contextual node information is used to predict whether or not toxicity will occur between drugs. This task is usually for drugs that are already in use and do not involve target information. There is no direct information about undiscovered drug–target pairs. However, the goal of this study was to predict and discover known and unknown drug–target pairs and the DDIs relationship can be used as only one of the aspects of calculating the similarity between drugs. PPIs focus on protein characteristics, such as the physiological functions of interactions between proteins, and can play an auxiliary role in discovering potential drugs [22]. PPIs are necessary for understanding most biological processes and are involved in most cellular activities. This task mainly uses sequence and structural information of proteins and uses only protein-related information without any drug information. This is insufficient to provide support and assistance for drug discovery. Therefore, PPIs are suitable only for providing one aspect of the protein similarity calculation necessary for drug discovery. In addition, DTA predicts successive values of the binding strength between drug targets, representing a more in-depth and detailed study of the nature of drug action [16, 21]. However, the contribution to the discovery of potential drug–target pairs is limited. DTA prediction is a regression task in which the output is a continuous measure of drug-target pair binding, typically using known drug-target pairs as training data, and then focusing on drug-target pairs that are known to interact and how strongly they interact. Drug-target pairs for which it is not yet known whether an interaction will occur are generally excluded from the training data for DTA prediction. However, this paper is precisely intended to focus on and address the discovery of potential unknown drug-target pairs. Therefore, in this study, we use the DTIs prediction task to provide direct information about potential drug–target pairs. This paper aims to train a model with known drug target pairs to mine some potential unknown drug-target pairs predictively. DTA is used to provide detailed information on known drug-target pairs, while DTIs prediction is used for known drug-target pairs and negative samples, i.e., drug-target pairs of potential screening value for which it is not yet certain if there is an interaction. Thus, the DTIs prediction task directly supports the ultimate goal of this paper.

Current machine learning methods for solving DTIs prediction can be categorized into the following six groups [23]: similarity matrix-based methods, deep learning methods, feature-based methods, matrix decomposition methods, network-based methods, and hybrid methods. Similarity matrix-based methods calculate proximity using different similarity scores or distance calculation formulas [24, 25]. The pharmacological similarity of drugs and genomic similarity of proteins can also be used to design similarity scoring schemes. In recent years, deep learning methods have been frequently applied to predict DTIs [26–28]. Such methods have been shown to have excellent performance in dealing with noisy data, such as high-dimensional data in drug repurposing. Deep learning methods use biological, physical, chemical, and network topology information of drugs and targets to generate feature vectors and train models using deep learning frameworks. However, deep learning models mostly lack stable and reliable negative samples during training, thus significantly reducing prediction performance. Feature-based methods include tree-based, kernel-based, and support vector machines, which represent drug and protein sequences with feature vectors of a certain length by constructing a specific feature space. Then, it builds various machine-learning models based on the feature representation to predict DTIs [29, 30]. Matrix decomposition methods involve decomposing the original observation matrix into two lower-order matrices [31, 32]. Matrix decomposition methods cannot depend on the chemical or pharmacological similarity of the drug because they use collaborative filtering algorithms that can minimize the error of point-by-point linear reconstruction of the dataset using low-rank embedded matrices. Network models are typically built by constructing heterogeneous networks [33–35] that integrate drug information, protein target information, and known DTIs, assuming that similar drugs act on similar targets. Hybrid methods effectively combine the above five methods to improve the algorithmic capability and robustness while mitigating the flaws and drawbacks of a single method.

Notably, most recent updates in the drug–target interactions field are network-related. Chatterjee et al. [36] proposed the AI-Bind model for DTIs prediction, which is based on a network sampling strategy that incorporates information on chemical molecular formulae of drugs and amino acid sequences of proteins and uses unsupervised learning to train the model. AI-Bind uses direct structural information, which is advantageous for generalizing to unseen drug–target pairs, but the predictive performance is insufficient. Zeng et al. [37] proposed the deepDTnet model for DTIs prediction. deepDTnet is a deep learning method that embeds multiple heterogeneous information from chemical, genomic, phenotypic, and cellular networks. Shang et al. [38] proposed a multilayer network representation called MEDTI for learning DTIs. The method demonstrated excellent performance in integrating multiple types of data and managing network noise. Wan et al. [39] proposed a nonlinear end-to-end learning model called NeoDTI, which can integrate a wide range of heterogeneous information and is robustly tolerant to hyperparameter selection. Li et al. [40] proposed an end-to-end collaborative contrast model called SGCL-DTI. SGCL-DTI can generate contrast losses to guide model optimization in a supervised manner. Most of these models rely on large datasets, and if the dataset is not large enough and does not cover enough information, the prediction performance will be significantly reduced. Therefore, the superiority of these models is partly due to the superiority of the dataset. We tried to apply the previous model to the same dataset as a baseline model for the algorithm proposed in this paper. In addition, we have chosen two knowledge graph link prediction models [41, 42] as baseline models for this paper. The above models are the baseline models because they are classical models through heterogeneous networks, knowledge graphs, etc., and are used by a wide range of scholars for discussion and comparison. It is objective to use them as a baseline for comparison.

This study uses a network method that mixes similarity computation and feature representation. The feature extraction and compression method is derived from an article published in Nature Communications by Luo et al. [43] in 2017. This study provides a framework for extracting compressed low-dimensional feature vectors from heterogeneous network information. In this work, we improve the network diffusion algorithm based on earlier research. The diffusion algorithm of the network is improved and upgraded in the feature extraction and network learning process, thereby improving the overall predictive efficacy of the model. In this paper, the random walk with restart (RWR) [44] was used to learn the structure of the network, and the Metropolis-Hasting random walk (MHRW) [45] algorithm was proposed to improve the network diffusion performance.

The MHRW algorithm draws on the Metropolis-Hasting process, which changes the traditional random walk algorithm’s strategy of equal probability toward neighboring nodes. The wandering particles generate different transfer probabilities using different network structures, which increases the comprehensiveness and accuracy of the algorithm in learning network structural features. We also applied the improved metropolis-hasting random walk (IMRWR) [46] algorithm to improve the performance of the MHRW. The algorithm removes the self-loop rate of the node so that the random walk particles are bound to walk to the next node in each iteration and will not stay in the same place. This improves the efficiency of network diffusion and promotes deep sampling. In this study, we propose a new network diffusion strategy by adding the self-loop probability of isolated nodes such that the wandering particles are more likely to perceive the isolated nodes rather than ignore them, avoiding wandering particles being trapped in dead ends. This method is named ISLRWR and is found to have excellent predictive performance in AUROC and AUPRC.

The features and advantages of this research can be summarized as follows.

(1) A combination of drug–drug, drug–disease, drug–side effect, protein–protein, protein–disease, and other heterogeneous network information is integrated as the original information for DTIs prediction.
(2) The MHRW and IMRWR algorithms were applied for DTIs network diffusion, and the performance was significantly improved compared to that of the RWR algorithm.
(3) The ISLRWR algorithm is proposed, and its performance is significantly improved based on the original model.
(4) Selecting deepDTnet, MEDTI, NeoDTI, SGCL-DTI, DistMult and ComplEx as baseline models, we used the same dataset to compare the DTIs prediction performance of ISLRWR and the baseline model. We found that ISLRWR performed well on a variety of evaluation indicators.

## Materials and methods

### Data presentation

The data used in this study consists of two main parts: drugs and proteins. The drug portion includes drug structural similarity, drug-related diseases, drug side effects, and drug–drug toxicity. The protein portion includes protein genomics similarity, protein-related diseases, and protein–protein interactions. In order to increase the objectivity of model performance comparison, we integrated two datasets for model training and evaluation, dataset A and dataset B, respectively. Drug data for dataset A were obtained from the Drugbank(3.0) database [47], the protein data were obtained from the HPRD database [48], the disease data were obtained from the CTD(2013) [49], and the side effect data were obtained from the SIDER (2.0) database [50]. In addition, drug data for dataset B were obtained from Drugbank(5.0) database [51], the protein data were obtained from the UniProt database [52], the disease data were obtained from the CTD(2021) database [53], and the side effect data were obtained from the SIDER (4.0) database [54]. In summary, we used four types of nodes (drug, disease, side-effect, and protein) and six types of edges (drug–drug, drug–disease, drug–side effect, drug–protein, protein–disease, and protein–protein) to construct the heterogeneous network. The node and edge statistics of dataset A and dataset B are presented in Tables 1 and 2.

**Table 1:** The node and edge statistics of dataset A.

Items	Type	Counts	Resources
Drug	Node	708	Drugbank(3.0) [47]
Protein	Node	1512	HPRD [48]
Disease	Node	5603	CTD(2013) [49]
Side-effect	Node	4192	SIDER(2.0) [50]
Drug-drug	Edge	10036	Drugbank(3.0) [47]
Drug-disease	Edge	199214	CTD(2013) [49]
Drug-side effect	Edge	80164	SIDER(2.0) [50]
Drug-protein	Edge	1923	Drugbank(3.0) [47]
Protein-protein	Edge	7363	HPRD [48]
Protein-disease	Edge	1596745	CTD(2013) [49]

**Table 2:** The node and edge statistics of dataset B.

Items	Type	Counts	Resources
Drug	Node	2214	Drugbank(5.0) [51]
Protein	Node	1968	UniProt [52]
Disease	Node	7205	CTD(2021) [53]
Side-effect	Node	3935	SIDER(4.0) [54]
Drug-drug	Edge	1091870	Drugbank(5.0) [51]
Drug-disease	Edge	542970	CTD(2021) [53]
Drug-side effect	Edge	104629	SIDER(4.0) [54]
Drug-protein	Edge	8750	Drugbank(5.0) [51]
Protein-protein	Edge	456592	UniProt [52]
Protein-disease	Edge	2922064	CTD(2021) [53]

### Overview of the model

The network model constructed for DTIs prediction can be decomposed into three parts: (1) constructing the heterogeneous network and importing the information; (2) calculating the drug similarity and protein similarity; and (3) extracting the compressed feature vectors through the network model, which uses random walk with restart as the diffusion algorithm to learn the topology of the network.

**Fig 1:**
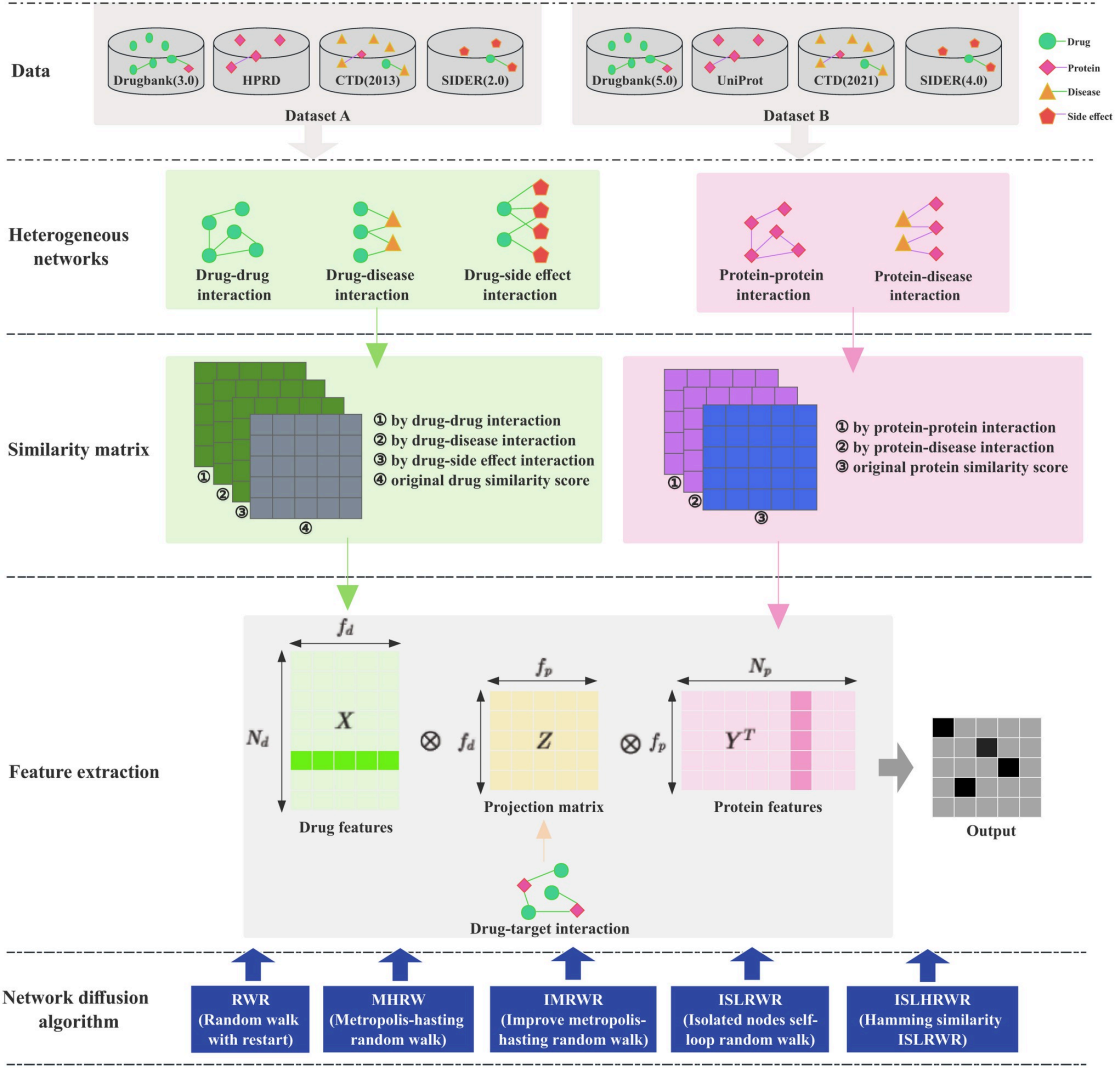
Overview of the network model. The green circles represent drug molecules, pink squares represent protein molecules, orange triangles represent diseases, and red pentagons represent side effects. The green and gray tables represent the drug similarity matrix and the purple and blue tables represent the protein similarity matrix. *N*_*d*_ indicates the number of drug nodes, *N*_*p*_ indicates the number of protein nodes, *f*_*d*_ indicates the length of the drug feature, and *f*_*p*_ indicates the length of the protein feature.

Fig 1 illustrates the framework structure of this paper. For the data part, we used two datasets consisting of four node types and six edge types respectively. The databases from which the node and edge data come from, respectively, are labeled in the Fig 1. The heterogeneous networks include five kinds of networks, three drug-related (drug-drug, drug–side effect, and drug–disease), and two protein-related (protein-protein and protein–disease). The similarity matrix was calculated according to drug and protein. In addition to the similarity information extracted from the heterogeneous network, we also supplemented the drug structure similarity and proteogenomic similarity. The feature extraction uses network diffusion algorithms to learn the network topology and uses the method proposed by Luo et al. [43] to obtain low-dimensional compressed feature vectors. We used the original restart random walk algorithm RWR [44]; the MHRW algorithm regarding the metropolis-hasting process [45]; the IMRWR algorithm with node self-loop rates removed [46]; the ISLRWR algorithm, which recalculates the transfer probabilities after adding the self-loop rates of the isolated nodes; and the ISLHRWR algorithm, which recomputes the similarity matrices. Then, we compared the five diffusion algorithms using experimental results. Note that the network diffusion algorithm is a specific subdivision algorithm of the feature extraction session so in the framework diagram “Network diffusion algorithm” directed “Feature extraction”. The green shading in Fig 1 indicates the flow and use of drug data, and the pink shading indicates the flow and use of protein data.

### Similarity calculations

In this paper, drug similarity is calculated using three heterogeneous networks related to drugs and supplemented with a matrix based on the structural similarity of drugs. Drug–drug interactions are represented by an adjacency matrix, where each element indicates whether the drug undergoes a toxic effect or other chemical reaction, where the presence of an element in 1 indicates an interaction. This drug similarity is based on the drug–drug interaction and is calculated using the Jaccard similarity coefficient, which focuses on the number and proportion of (1,1) tuples in the two 0-1 vectors. Similarly, we used the drug–disease interaction network to obtain the drug similarity matrix using the disease as a benchmark, where drugs acting on the same disease are considered to have some degree of similarity. Based on the drug-side effect interaction network, we obtained a similarity matrix comparing the similarities and differences and the degree of crossover of drug side effects. An additional drug structural similarity matrix was added to obtain four dimensions of drug based similarity. We calculated a drug structural similarity using the drug’s SMILES (simplified molecular-input line-entry system) sequence. We used Tanimoto scores [55] to obtain the drug’s structural similarity.

Similarly, a similarity matrix was obtained based on protein–protein interactions, another similarity matrix was obtained using disease as a comparison principle through the protein–disease interaction network, and a protein genome similarity matrix was supplemented, such that there were three matrices for protein similarity. We calculated the protein genome similarity from the amino acid sequence of proteins. We calculated the normalized Smith-Waterman score [56] as the genomic similarity of the proteins.

The Jaccard similarity coefficients were calculated using the Eq (1).

$$\begin{array}{c} J\left(i,j\right)=\frac{M_{11}}{M_{11}+M_{01}+M_{10}} \end{array}$$ (1)

**Fig 2:**
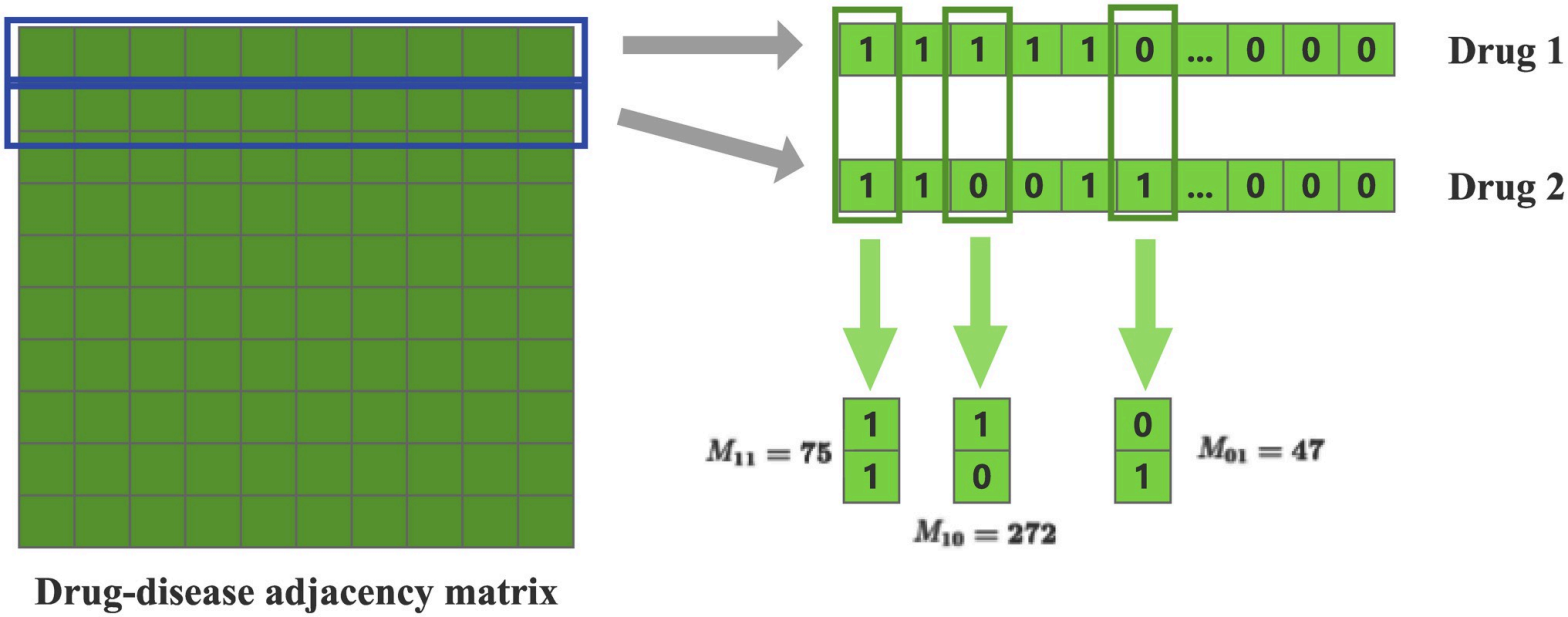
Similarity calculation process.

J(i,j) denotes the Jaccard similarity of drug i and drug j. *M*_11_ denotes the number of (1,1) tuples of 0-1 vectors of drug i and drug j responding simultaneously to 1. *M*_01_ denotes the number of (0,1) tuples for drug i and drug j. *M*_10_ denotes the number of (1,0) tuples for drugs i and j. For example, in the drug-disease adjacency matrix, the row vector of *drug*_1_ is [1, 1, 1, 1, 1, 0, …, 0, 0, 0]_1*5603_, and the row vector of *drug*_2_ is [1, 1, 0, 0, 1, 1, …, 0, 0, 0]_1*5603_. The number of (1,1) tuples is 75,the number of (1,0) tuples is 272 and the number of (0,1) tuples is 47. Then the similarity between *drug*_1_ and *drug*_2_ is calculated as: $\frac{75}{75+272+47}=0.19$. We show the computational process as Fig 2.

### Low-dimensional feature extraction

This network feature extraction method [43] can handle highly noisy, incomplete, and large-scale high-dimensional biological data to obtain a low-dimensional and informative vector representation. The method learns contextual information in a single network and topological properties in multiple networks. Based on the obtained low-dimensional vector representation, the best projection from drug space to target space can be found, thus predicting a new DTIs based on the geometric proximity of the projection mapping.

As shown in Fig 1, the X- and Y-features of the drug and protein are learned, where *N*_*d*_ denotes the number of drug nodes, *N*_*p*_ denotes the number of protein nodes, *f*_*d*_ denotes the length of the drug features, and *f*_*p*_ denotes the length of the protein features. The optimal projection matrix Z is then found to minimize the difference between the interaction matrix and *XZY*_*T*_. The resulting low-dimensional feature vectors encode relational attributes (e.g., similarity), association information, and the topological context of the drug and protein nodes.

### Network diffusion algorithm

The diffusion state of the network is learned using the restarted random walk algorithm, which obtains a low-dimensional feature vector representation of a single node by minimizing the difference between the diffusion state and a parameterized polynomial logic model. The transfer probability of the diffusion state of the network is first computed using the original RWR [44]. As shown in Eq (2), *K*(*i*) is the degree of node *i*, $P_{ij}^{\left(1\right)}$ is the transfer probability matrix calculated by the RWR algorithm. Γ(*i*) denotes the set of neighboring nodes to which node *i* is connected. *v*_*j*_ denotes the next node.

$$\begin{array}{c} P_{ij}^{\left(1\right)}=\{\begin{array}{c} \frac{1}{K(i)},v_{j}\in \Gamma \left(i\right)\\ 0,others \end{array} \end{array}$$ (2)

Then, assuming that the probability of a particle randomly going to the next node is c, and the probability of returning to the initial node is 1—c, the probability vector of a particle reaching each node in the network is Eq (3).

$$\begin{array}{c} \pi_{i}\left(t+1\right)=cP^{T}\pi_{i}\left(t\right)+\left(1-c\right)e_{i} \end{array}$$ (3)

As shown in Eq (3), *e*_*i*_ is the start vector, *e*_*i*_ = 1 if *i* is the start node, otherwise *e*_*i*_ = 0. *π*_*i*_ is the column vector, *π*_*i*_(*t*) denotes the probability vector of other nodes transferring to node *i* at moment *t*. *π*_*i*_(*t* + 1) denotes the probability vector of other nodes transferring to node *i* at moment *t* + 1. Then the steady-state solution of Eq (3) is Eq (4).

$$\begin{array}{c} \pi_{i}=\left(1-c\right){\left(I-cP^{T}\right)}^{-1}e_{i} \end{array}$$ (4)

In addition, we used the MHRW algorithm [45], which is borrowed from the metropolis–hasting process, and the transfer probability is calculated as Eq (5).

$$\begin{array}{c} P_{ij}^{\left(2\right)}=\{\begin{array}{c} \frac{1}{K(i)}\;\text{min}\;(1,\frac{K(i)}{K(j)}),v_{j}\in \Gamma \left(i\right)\\ 1-\sum_{l}p_{il}v_{l}=v_{i},v_{l}\in \Gamma \left(i\right)\\ 0,\;\text{others} \end{array} \end{array}$$ (5)

As shown in Eq (5), *K*(*i*) is the degree of node *i*, *K*(*j*) is the degree of node *j*. $P_{ij}^{\left(2\right)}$ is the transfer probability matrix calculated by the MHRW algorithm. Γ(*i*) denotes the set of neighboring nodes to which node *i* is connected. *v*_*j*_,*v*_*l*_ denotes the nodes in the Γ(*i*). *p*_*il*_ denotes the transfer probability value from node *i* to node *l*. Accordingly, we also applied the IMRWR [46] algorithm to calculate the transfer probability matrix, as Eq (6). $P_{ij}^{\left(3\right)}$ is the transfer probability matrix calculated by the IMRWR algorithm.

$$\begin{array}{c} P_{ij}^{\left(3\right)}=\{\begin{array}{c} \frac{1}{K(i)}min\left(1,\frac{K(i)}{K(j)}\right)+\frac{K(j)}{\sum_{l}K\left(l\right)}P_{ii}^{\left(2\right)},v_{j},v_{l}\in \Gamma \left(i\right)\\ 0,others \end{array} \end{array}$$ (6)

Finally, we proposed the ISLRWR algorithm for recalculating the transfer probability of particles after adding the self-loop probability of isolated nodes. As shown in Eq (7), $P_{ij}^{\left(4\right)}$ is the transfer probability matrix calculated by the ISLRWR algorithm. Γ(*i*) denotes the set of neighboring nodes to which node *i* is connected. *v*_*j*_ denotes the next node. *n*_*i*_ denotes the number of isolated nodes. *J*(*i*, *j*) denotes similarity between node *i* and node *j*.

$$\begin{array}{c} P_{ij}^{\left(4\right)}=\{\begin{array}{c} \frac{J(i,j)}{\sum_{j}J\left(i,j\right)+n_{i}},v_{j}\in \Gamma \left(i\right)\\ 0,others \end{array} \end{array}$$ (7)

**Fig 3:**
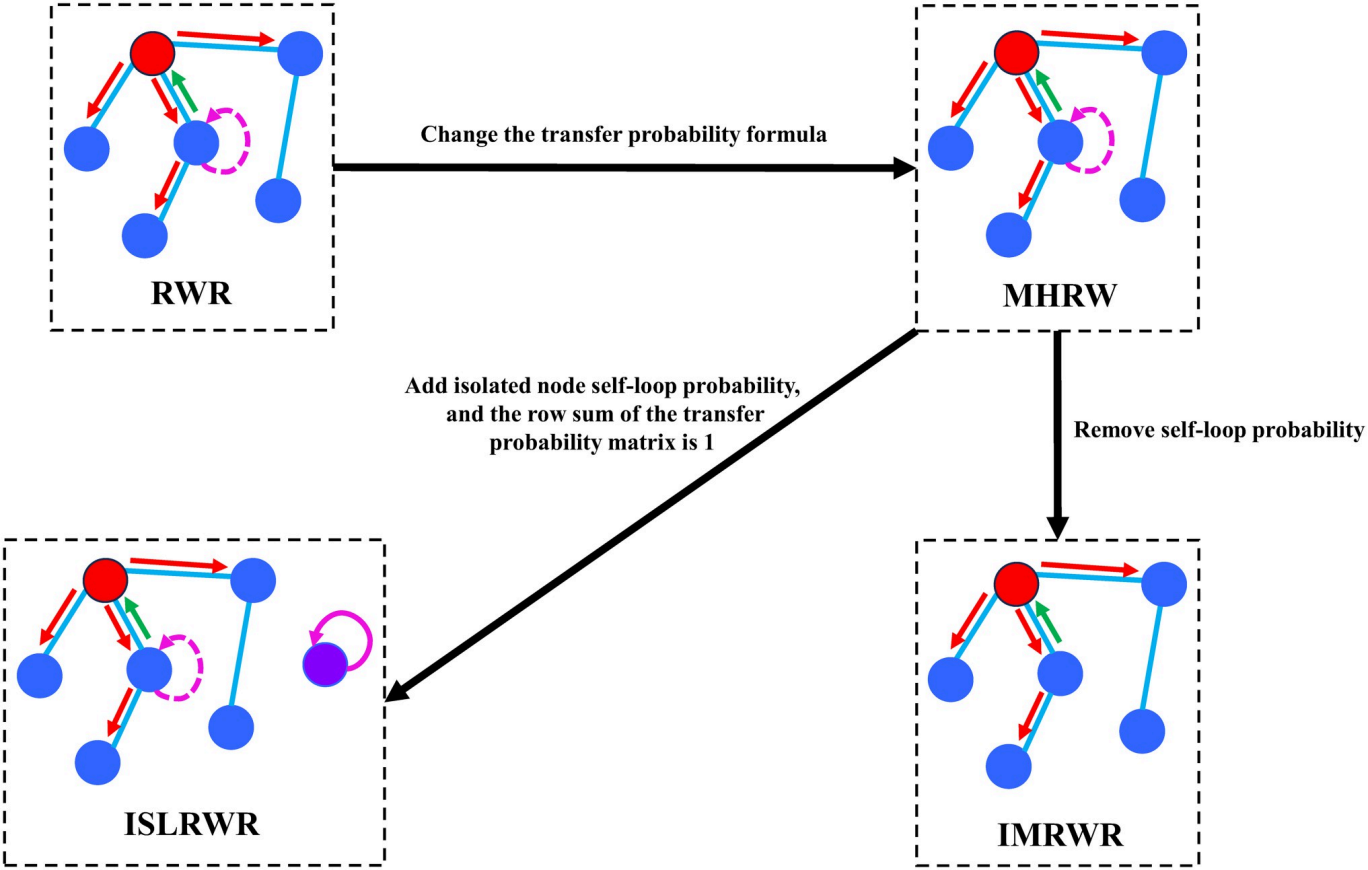
Schematic of the random walk algorithm. The red arrow in the figure indicates that the particle goes to the next step, the green arrow indicates that the particle returns to the previous step, and the pink arrow indicates that the particle stays at the current node.

The evolution of the above random walk diffusion algorithm is shown in Fig 3, which visualizes the optimization of the transfer probability and the change in the sampling strategy of the MHRW algorithm compared to the RWR algorithm. The IMRWR removes the self-loop probability of the current node compared to the MHRW algorithm, which ensures that the wandering particles transfer at each step and do not stay in the same place. The ISLRWR adds the self-loop probability of isolated nodes and then recalculates the current node’s transfer probability, ensuring that the row sum of the matrix of transfer probabilities is 1.

### Baseline models and evaluation metrics

We chose MEDTI, deepDTnet, SGCL-DTI, DistMult, ComplEx, and NeoDTI as the baseline models because these models have attracted much attention in recent years in the research of related fields and not only have gained extensive discussion and high evaluation but also their reliability has been fully verified. They have demonstrated remarkable innovativeness at the technical level, providing new perspectives and solutions for research in the field of DTIs prediction by utilizing advanced algorithms and unique architectures. Therefore, we select these models as a baseline for comparison, aiming to assess the performance of the models proposed in this paper more comprehensively through in-depth comparison and analysis so as to reveal their strengths and weaknesses and provide valuable references and insights for future research work.

We used six different evaluation metrics to comprehensively assess the performance of the model, which are precision score, recall score, F1 score, matthews correlation coefficient (MCC), area under the receiver operating characteristic curve (AUROC), and area under the precision-recall curve (AUPRC). In binary classification problems, we can usually obtain counts of true negatives (TN), false negatives (FN), true positives (TP), and false positives (FP). Among them, precision score is derived by calculating the ratio *TP*/(*TP* + *FP*), which reflects the ability of the classifier to avoid incorrectly labeling negative samples as positive. Recall score, on the other hand, is derived by calculating the ratio *TP*/(*TP* + *FN*), which measures the classifier’s ability to find all positive samples. The F1 score is the reconciled mean of precision and recall, and is calculated as *F*1 = 2 * (*precision* * *recall*)/(*precision* + *recall*). In addition, MCC as a measure of the quality of binary categorization has a value range of -1 to +1 and is usually considered as a balanced measure. Except for MCC, the values of the other evaluation metrics lie between 0 and 1.

## Results

### Evaluation of the drug-target interaction predicting capabilities

In this study, ten-fold cross-validation was used to train and evaluate the model by randomly dividing all known DTIs (known positive samples) into ten equal portions, selecting one of them at a time, and randomly sampling the same number of non-interacting drug–target pairs (the same number of negative samples generated) as the test set. The remaining 90% of the positive samples with known interactions were used as the training set, along with the same number of negative samples generated.

In the DTIs prediction task, drug–target pairs with known interactions were available from databases. We obtained samples with unknown interactions from random sampling matches from the drug list and protein list. In most cases, we assumed that drug–target pairs obtained by random sampling were free of interaction situations unless there was preexisting literature on the subject or it had been stored in a database. The approach taken in this study was to assign label 0 to drug–target pairs obtained by random matching and label 1 to drug–target pairs with known interactions obtained from the database. Note that the number of samples for label 0 was the same as the number of samples for label 1. If the negative sample (label 0) obtained from the sampling was found to be already present in the positive list, then we deleted the negative sample and resampled the sampling until the number of negative and positive samples (label 1) were equal.

**Fig 4:**
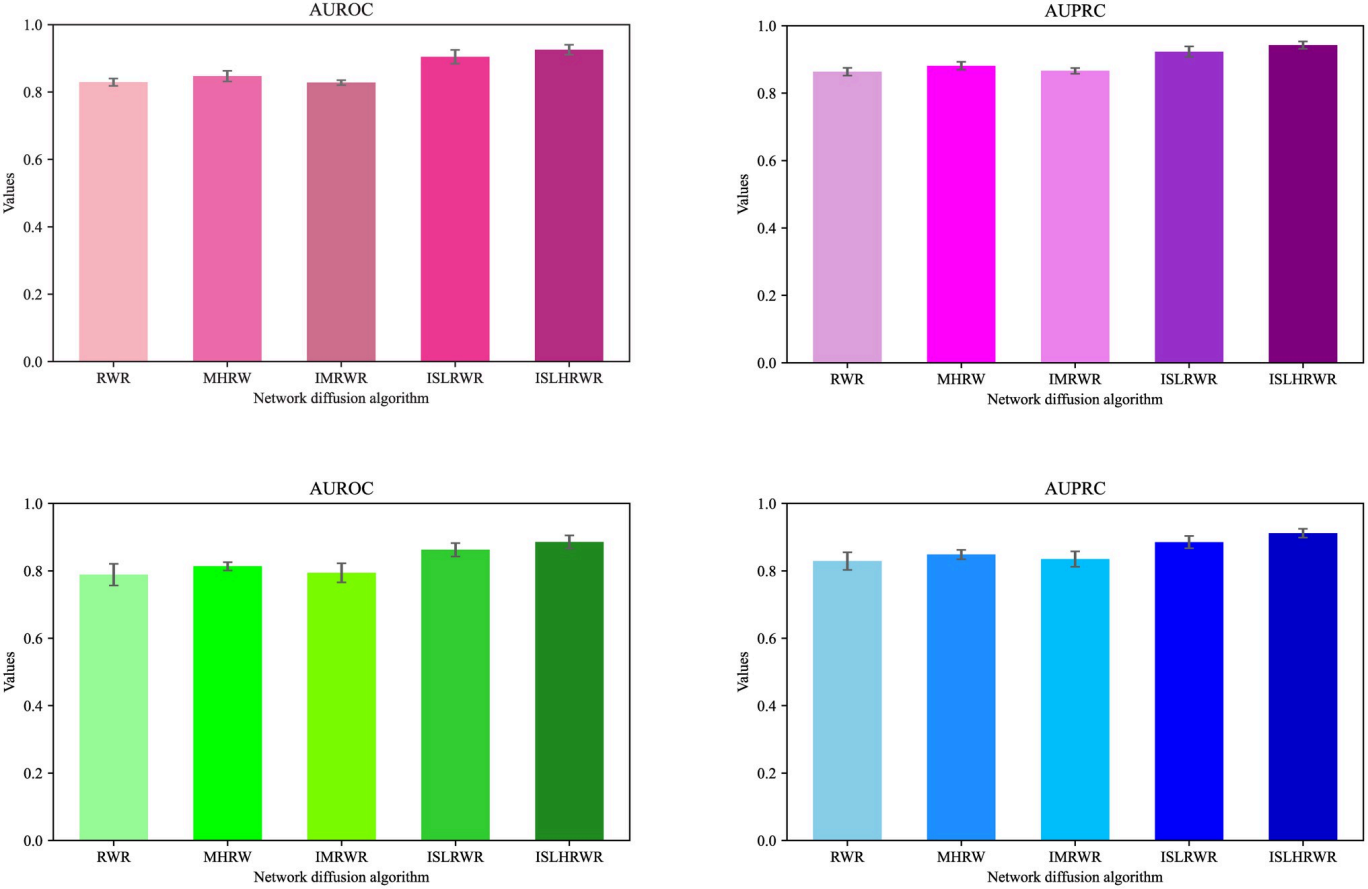
Performance of network diffusion algorithms. The graphs indicate the high and low values of the evaluation indicators. The darker the color the higher the value. The top short line indicates the standard deviation of the ten-fold cross-validation.

We calculated the mean and standard deviation of the AUROC and AUPRC for the ten crossover trials, as shown in Table 3. To exclude the interference of homologous proteins, drug–target pairs with protein sequence identity scores of 0.4, as well as drug similarity of 0.6 or more, were removed. Because popular and commonly used drugs and proteins are more likely to be predicted, which may lead to inflated and potentially redundant predictions, we deleted these data and reassessed the above methodology. In this study, the enhancement effects of the AUROC and AUPRC calculated from the above cases are visualized in Fig 4, a bar chart containing short standard deviations.

**Table 3:** Performance of different network diffusion algorithms.

Model	Ordinary		Removal of homologous proteins	
	AUROC	AUPRC	AUROC	AUPRC
RWR	0.8290 ± 0.0110	0.8637 ± 0.0113	0.7888 ± 0.0322	0.8292 ± 0.0262
MHRW	0.8471 ± 0.0160	0.8813 ± 0.0118	0.8136 ± 0.0124	0.8483 ± 0.0138
IMRWR	0.8277 ± 0.0073	0.8662 ± 0.0083	0.7942 ± 0.0284	0.8350 ± 0.0226
ISLRWR	0.9043 ± 0.0204	0.9232 ± 0.0154	0.8626 ± 0.0198	0.8852 ± 0.0182
ISLHRWR	0.9254 ± 0.0147	0.9425 ± 0.0110	0.8857 ± 0.0196	0.9118 ± 0.0128

The MHRW algorithm improves the performance of learning the network structure and predicting the DTIs over the original RWR algorithm; the AUROC improves by 1.81%, and the AUPRC improves by 1.76%. In contrast, the IMRWR algorithm does not show a significant performance improvement. The ISLRWR algorithm improved AUROC by 5.72% and AUPRC by 4.19% compared with the MHRW algorithm. The ISLRWR algorithm improved AUROC by 7.53% and AUPRC by 5.95% compared with the RWR algorithm. This result showed that the prediction performance of the ISLRWR algorithm improved significantly. In addition, the ISLHRWR algorithm uses the Hamming similarity calculation method to recalculate the similarity matrix and then uses ISLRWR to learn the network structure to obtain better prediction performance. The ISLHRWR algorithm improves the AUROC and AUPRC by 2.11% and 1.93%, respectively, compared to the ISLRWR algorithm.

In addition, the performance improvement of the above algorithms still exists after removing homologous proteins. The MHRW algorithm outperforms the RWR algorithm with a 2.48% improvement in AUROC and a 1.91% improvement in AUPRC. The IMRWR algorithm improves the AUROC and AUPRC by 0.54% and 0.58%, respectively, compared to the RWR algorithm. The ISLRWR algorithm improves the AUROC and AUPRC by 4.90% and 3.69%, respectively, compared to the MHRW algorithm, which improves the AUROC and AUPRC by 7.38% and 5.60%, respectively, compared to the RWR algorithm. The ISLHRWR algorithm improves the AUROC and AUPRC by 2.31% and 2.66%, respectively, compared to the ISLRWR algorithm. The removal of homologous proteins alleviates the problem of inflated prediction results to some extent.

**Fig 5:**
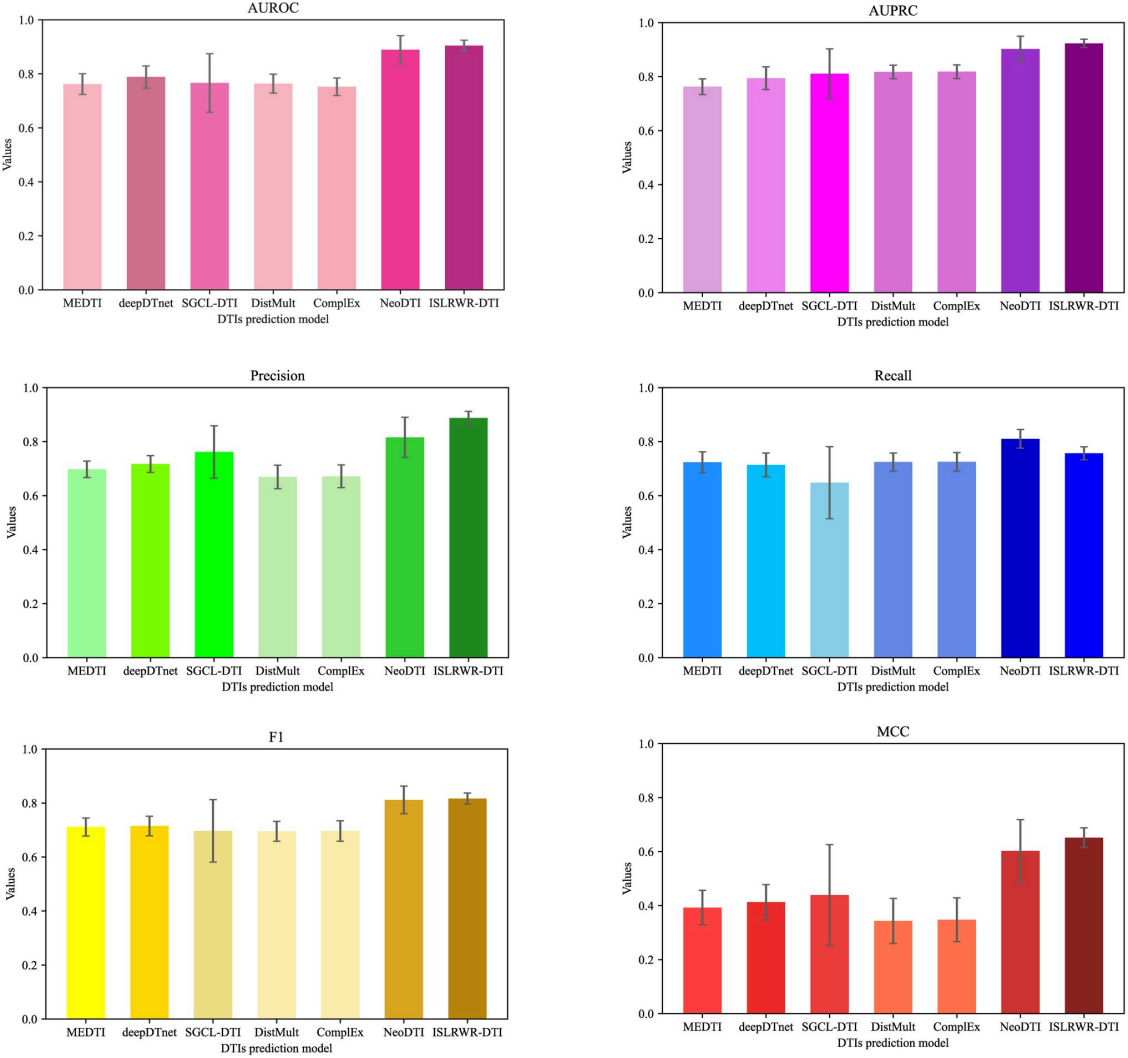
Comparison of models based on dataset A. The graphs indicate the high and low values of the evaluation indicators. The darker the color, the higher the value. The top short line indicates the standard deviation of the ten-fold cross-validation.

**Fig 6:**
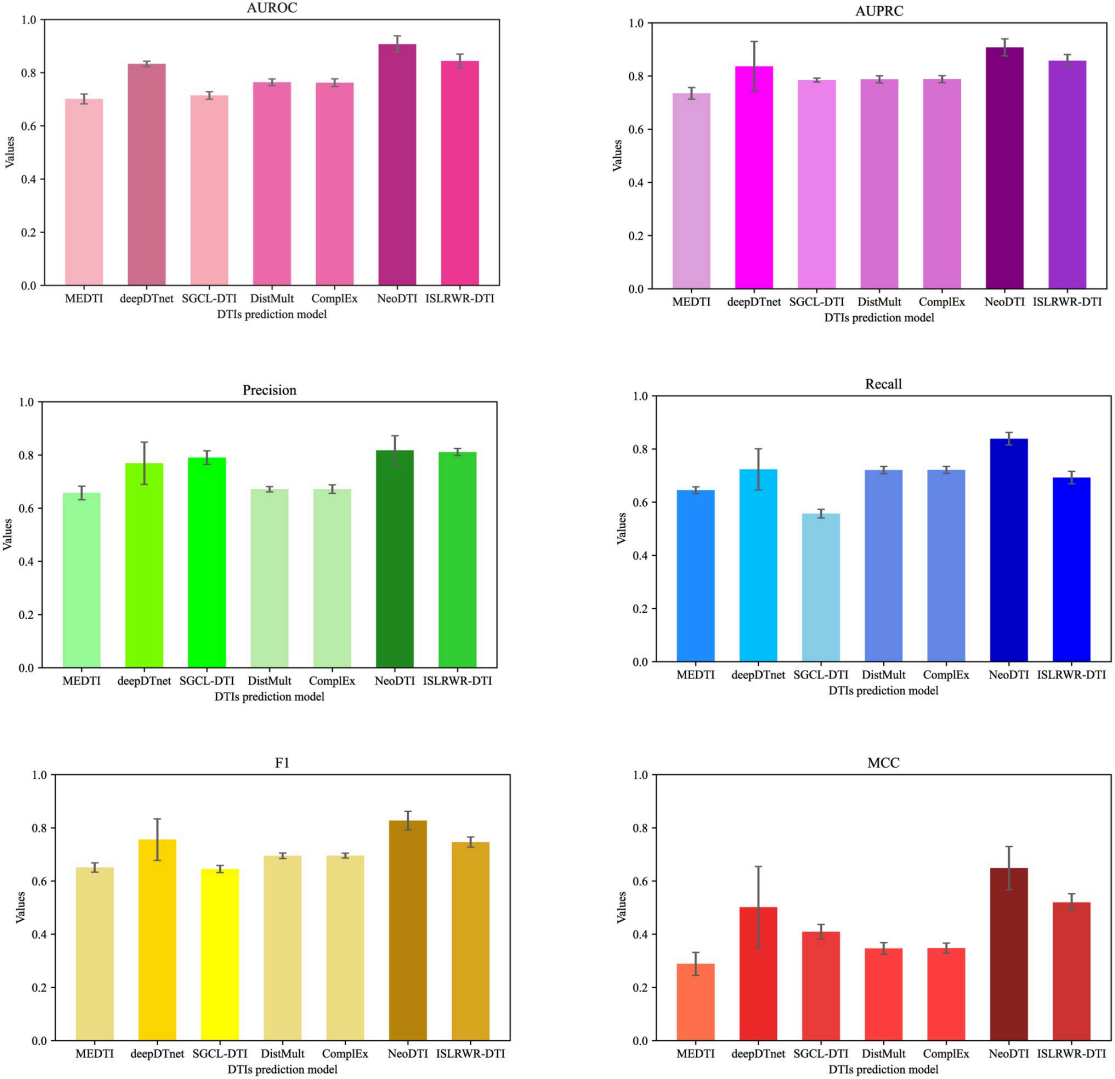
Comparison of models based on dataset B. The graphs indicate the high and low values of the evaluation indicators. The darker the color, the higher the value. The top short line indicates the standard deviation of the ten-fold cross-validation.

To comprehensively demonstrate the performance of ISLRWR in DTIs prediction, we selected MEDTI, deepDTnet, SGCL-DTI, DistMult, ComplEx and NeoDTI as baseline models to compare them with ISLRWR. We choose AUROC, AUPRC, precision score, recall score, F1 score, and MCC as evaluation indicators. We compared ISLRWR-DTI with the baseline model on dataset A and found that ISLRWR performed the best on AUROC, AUPRC, precision score, F1 score and MCC. Regarding recall score, however, NeoDTI was better than ISLRWR. In addition we compared ISLRWR-DTI with the baseline model on dataset B as well. We find that deep learning models like DEEP and NeoDTI perform better on large-scale datasets, but perform weakly when the amount of data is not large enough. The results based on dataset A are presented in Table 4 and Fig 5. The results based on dataset B are presented in Table 5 and Fig 6. In summary, the prediction performance of ISLRWR-DTI is still superior on small and medium sized datasets and is slightly inferior but acceptable on large datasets.

**Table 4:** Comparison of ISLRWR-DTI with baseline models on dataset A.

Models	AUROC	AUPRC	Precision	Recall	F1	MCC
MEDTI	0.7621 ± 0.0382	0.7628 ± 0.0291	0.6976 ± 0.0304	0.7236 ± 0.0384	0.7113 ± 0.0335	0.3926 ± 0.0642
deepDTnet	0.7883 ± 0.0412	0.7942 ± 0.0420	0.7170 ± 0.0311	0.7138 ± 0.0443	0.7152 ± 0.0362	0.4128 ± 0.0651
SGCL-DTI	0.7662 ± 0.1083	0.8110 ± 0.0916	0.7615 ± 0.0972	0.6480 ± 0.1334	0.6968 ± 0.1157	0.4393 ± 0.1864
DistMult	0.7636 ± 0.0351	0.8175 ± 0.0252	0.6692 ± 0.0435	0.7246 ± 0.0334	0.6954 ± 0.0367	0.3432 ± 0.0835
ComplEx	0.7521 ± 0.0326	0.8182 ± 0.0254	0.6715 ± 0.0421	0.7251 ± 0.0342	0.6965 ± 0.0378	0.3476 ± 0.0813
NeoDTI	0.8890 ± 0.0524	0.9021 ± 0.0475	0.8159 ± 0.0741	0.8104 ± 0.0346	0.8118 ± 0.0512	0.6025 ± 0.1160
ISLRWR-DTI	0.9043 ± 0.0204	0.9232 ± 0.0154	0.8877 ± 0.0244	0.7569 ± 0.0241	0.8167 ± 0.0203	0.6516 ± 0.0362

**Table 5:** Comparison of ISLRWR-DTI with baseline models on dataset B.

Models	AUROC	AUPRC	Precision	Recall	F1	MCC
MEDTI	0.7013 ± 0.0185	0.7353 ± 0.0218	0.6575 ± 0.0254	0.6450 ± 0.0127	0.6511 ± 0.0173	0.2887 ± 0.0434
deepDTnet	0.8329 ± 0.0102	0.8365 ± 0.0937	0.7692 ± 0.0793	0.7234 ± 0.0774	0.7560 ± 0.0781	0.5016 ± 0.1531
SGCL-DTI	0.7145 ± 0.0142	0.7849 ± 0.0075	0.7906 ± 0.0256	0.5569 ± 0.0162	0.6455 ± 0.0133	0.4095 ± 0.0274
DistMult	0.7641 ± 0.0125	0.7877 ± 0.0133	0.6714 ± 0.0103	0.7211 ± 0.0136	0.6953 ± 0.0104	0.3468 ± 0.0217
ComplEx	0.7621 ± 0.0146	0.7882 ± 0.0135	0.6719 ± 0.0162	0.7215 ± 0.0132	0.6957 ± 0.0091	0.3477 ± 0.0192
NeoDTI	0.9072 ± 0.0314	0.9078 ± 0.0321	0.8176 ± 0.0552	0.8387 ± 0.0237	0.8272 ± 0.0352	0.6488 ± 0.0812
ISLRWR-DTI	0.8439 ± 0.0261	0.8578 ± 0.0234	0.8112 ± 0.0132	0.6927 ± 0.0235	0.7465 ± 0.0193	0.5196 ± 0.0327

### Noteworthy drug-protein pair analysis

In summary, the ISLRWR algorithm has the best performance in learning network structure and predicting DTIs compared to the RWR, MHRW, and IMRWR algorithms. Therefore, to obtain more auxiliary information for drug screening through the prediction results, we discuss the common networks in the DTIs network, focusing on the six drug–target common networks that are most densely connected with drugs. We determined the density of the shared network by degree. The higher the number of shared drugs between two targets, the denser the network. We selected the six dense networks with the highest number of shared connections for interpretation.

**Fig 7:**
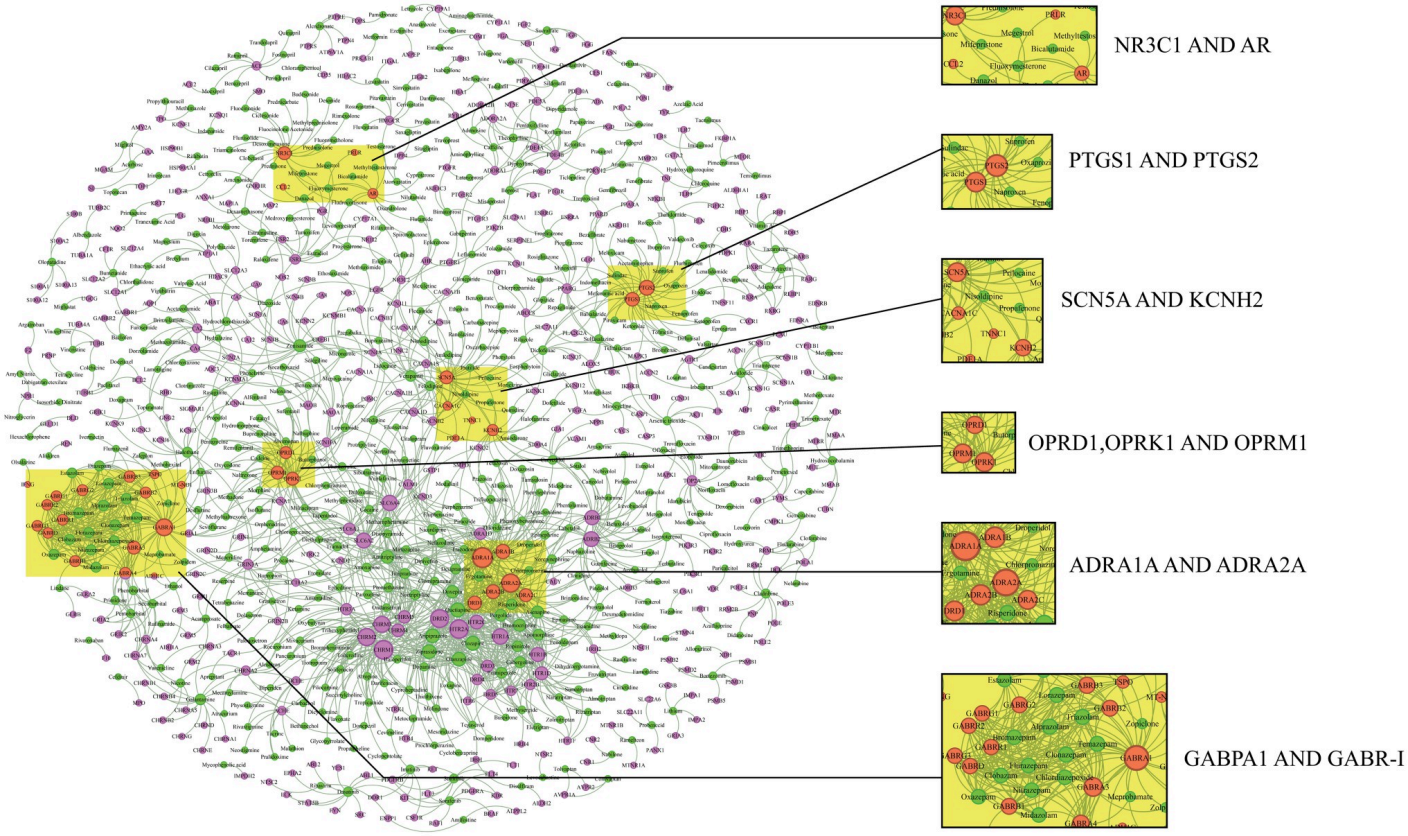
Drug–target interaction network diagram. The green nodes indicate drugs and the pink nodes indicate targets. Mixed-color arc connections represent node–node interactions. The yellow shadows are used to highlight the network communities that require attention.

According to Fig 7, fludrocortisone and fluoxymesterone are in the shared network of target NR3C1 and target AR. The efficacy of the drugs in the sharing network is summarized in Table 6.

**Table 6:** Drug efficacy in the shared network of targets NR3C1 and AR.

Drug	Efficacy
Fludrocortisone	Corticosteroids with anti-inflammatory and anti-allergic effects
Fluoxymesterone	Treatment of muscle atrophy and muscle damage

The network of the two homologous targets of PTGS1 and PTGS2 contains 22 associated drugs, the main effects of which are antipyretic and anti-inflammatory. Antipyretic and analgesic drugs include acetaminophen, indomethacin, napumetone, ketorolac, tolmetin, piroxicam, fenoprofen, diclofenac, mefenamic acid, naproxen, mexicam, diflunisal, suprofen, bromfenac, balsalazide, and ibuprofen. In addition, the following drugs are commonly used to treat rheumatoid arthritis: Sulindac, flurbiprofen, etodolac, sulfasalazine, oxaprozin, and ketoprofen.

Three drugs are co-associated with the targets SCN5A and KCNH2. The efficacy of the drugs in the sharing network is summarized in Table 7.

**Table 7:** Drug efficacy in the shared network of targets SCN5A and KCNH2.

Drug	Efficacy
Verapamil	Treats hypertension, coronary heart disease, and arrhythmia
Quinidine	Antiarrhythmics for atrial fibrillation and atrial flutter
Propafenone	Broad-spectrum and highly potent membrane-inhibitory antiarrhythmic drugs

Sixteen drugs are associated with the targets OPRD1 (gene for *μ* -opioid receptor), OPRK1, and OPRM1 (*μ*-opioid receptor). The efficacy of the drugs in the sharing network is summarized in Table 8.

**Table 8:** Drug efficacy in the shared network of targets OPRD1, OPRK1 and OPRM1.

Drug	Efficacy
Tramadol	Codeine analogs, performing analgesic effects
Morphine	Opioid agonists, commonly used as clinical anesthetics
Codeine	Opioids used to treat mild to moderate pain
Hydromorphone	Semi-synthetic derivatives of morphine
Oxycodone	Semi-synthetic derivative of Tibaine, potent analgesic
Butorphanol	Treatment of various types of cancer pain, including post-surgical pain
Naltrexone	Opioid receptor pure antagonist
Sufentanil	Used as an analgesic
Fentanyl	Potent opioid painkillers
Loperamide	Long-acting anti-diarrhea drugs
Nalbuphine	Powerful painkiller
Remifentanil	Ultrashort-acting *μ*-opioid agonists
Buprenorphine	Opioid receptor partial agonist
Naloxone	Morphine receptor antagonist
Tapentadol	Opiate analgesic
Alvimopan	Opioid *μ*-receptor inhibitors

Twenty-three drugs are associated with ADRA1A and ADRA2A. Ziprasidone is an atypical antipsychotic. Amitriptyline is a depression medication that is used to treat all types of depression or to relieve chronic pain. Olanzapine is an atypical neuroleptic. In addition, the seven drugs in this co-association network, including ziprasidone, amitriptyline, olanzapine, clozapine, doxepin, quetiapine, and aripiprazole, were also associated with HTR2A and CHRM1.

GABPA1 is in a shared network with GABR-i and other targets. Alprazolam is a benzodiazepine hypnotic sedative and anxiolytic agent. Chlordiazepoxide has sedative, anxiolytic, muscle relaxant, and anticonvulsant effects. Midazolam is used clinically for treating insomnia and can also be used to induce sleep during surgical procedures. In addition, flurazepam, diazepam, oxazepam, triazolam, clonazepam, estazolam, bromazepam, and nitrazepam can be used to induce sedative and tranquilizing effects.

Furthermore, we enumerate some of the correctly predicted drug–target pairs to provide supporting information for drug discovery. We selected three drugs Vitamin A, Eletriptan, and Olanzapine with the targets that interact with them as examples. The predicted results are given in Table 9.

**Table 9:** Prediction results of three drugs Vitamin A, Eletriptan and Olanzapine.

Drug ID : Drug name	Target ID : Target name	Prediction result
DB00162 : Vitamin A	O95237 : LRAT	True
DB00162 : Vitamin A	P00352 : ALDH1A1	True
DB00162 : Vitamin A	P09455 : RBP1	True
DB00162 : Vitamin A	P10745 : RBP3	True
DB00162 : Vitamin A	P12271 : RLBP1	True
DB00162 : Vitamin A	Q92781 : RDH5	True
Accuracy		100%
DB00216 : Eletriptan	P28221 : HTR1D	True
DB00216 : Eletriptan	P28222 : HTR1B	True
DB00216 : Eletriptan	P30939 : HTR1F	True
DB00216 : Eletriptan	P34969 : HTR7	True
DB00216 : Eletriptan	P41595 : HTR2B	True
Accuracy		100%
DB00334 : Olanzapine	P08172 : CHRM2	True
DB00334 : Olanzapine	P08173 : CHRM4	True
DB00334 : Olanzapine	P08908 : HTR1A	True
DB00334 : Olanzapine	P08912 : CHRM5	True
DB00334 : Olanzapine	P08913 : ADRA2A	True
DB00334 : Olanzapine	P11229 : CHRM1	True
DB00334 : Olanzapine	P14416 : DRD2	True
DB00334 : Olanzapine	P18089 : ADRA2B	True
DB00334 : Olanzapine	P18825 : ADRA2C	True
DB00334 : Olanzapine	P20309 : CHRM3	True
DB00334 : Olanzapine	P21728 : DRD1	True
DB00334 : Olanzapine	P21917 : DRD4	True
DB00334 : Olanzapine	P21918 : DRD5	True
DB00334 : Olanzapine	P28221 : HTR1D	True
DB00334 : Olanzapine	P28222 : HTR1B	False
DB00334 : Olanzapine	P28223 : HTR2A	True
DB00334 : Olanzapine	P28335 : HTR2C	True
DB00334 : Olanzapine	P34969 : HTR7	True
DB00334 : Olanzapine	P35348 : ADRA1A	True
DB00334 : Olanzapine	P35368 : ADRA1B	True
DB00334 : Olanzapine	P35462 : DRD3	False
DB00334 : Olanzapine	P46098 : HTR3A	True
DB00334 : Olanzapine	P50406 : HTR6	True
Accuracy		91.30%

In addition, by carefully screening the prediction results, we obtained the following newly discovered potential drug–target pairs. We defined a new drug–target pair as a drug ID and target ID, which are both searchable in the original training data. The drug–target pair was not known to exist in the training data but was predicted to be true in the subsequent prediction results. We obtained such drug–target pairs from sampling matches between the pre-drug ID list and the target ID list. We put such results into currently published popular drug databases for searching to verify whether such findings were true and reliable. Some of the new drug–target pairs verified as true by the database searches are displayed in Table 10.

**Table 10:** Database search support for new drug target pairs.

Drug ID : Drug name	Target ID : Target name	Database
DB00829 : Diazepam	P48169 : GABRA4	DrugBank [51]
DB01215 : Estazolam	P48169 : GABRA4	DrugBank [51]
DB00363 : Clozapine	P21918 : DRD5	DrugCentral [57]
DB06216 : Asenapine	P28221 : HTR1D	DrugCentral [57]
DB00696 : Ergotamine	P08908 : HTR1A	DrugBank [51], DrugCentral [57]
DB00543 : Amoxapine	P28223 : HTR2A	DrugBank [51], DrugCentral [57]

## Discussion

Traditional drug discovery methods are both time-consuming and costly; therefore, it is valuable to use computer-aided techniques such as machine learning methods to improve the efficiency of drug discovery. Many machine learning methods have been applied to various aspects of drug discovery, among which, DTIs prediction is an important facilitating task for discovering new drugs and potential targets.

However, many of the DTIs models rely on large datasets, and if the dataset is not large enough and does not cover enough information, the prediction performance will be greatly reduced. In other words, the superiority of these models is partly due to the superiority of the dataset. In this study, we focused on the model and the algorithm itself to improve the prediction performance. Few scholars have paid attention to net diffusion algorithms for network feature extraction. We applied the MHRW and IMRWR algorithms and proposed the ISLRWR algorithm. We found that by improving the network diffusion strategy, we could improve the prediction performance of DTIs to some extent.

In this study, we integrate four kinds of nodes (drug, target, disease, and side-effect) and six kinds of edges (drug-drug, drug–side effect, drug-disease, drug-protein, protein-protein, and protein–disease) as heterogeneous networks. Then, we learn the network topology using multiple network diffusion algorithms to obtain compressed low-dimensional feature vectors. We applied the MHRW algorithm to improve the diffusion performance of the RWR algorithm. Further, we applied the IMRWR algorithm to remove the self-loop rate of the current node to improve the efficiency and sampling depth of network learning. In summary, we propose an ISLRWR algorithm that increases the self-loop rate of isolated nodes and then re-corrects the transfer probability matrix, significantly improving the AUROC and AUPRC of DTIs prediction. There is potential for data inflation phenomenon, i.e., an excessive number of homologous proteins leads to popular drugs and targets being predicted more easily and with a notable prediction tendency, resulting in non-objective prediction results. Therefore, homologous proteins were removed, and the above algorithm was applied again, revealing that the ISLRWR still had excellent performance. To comprehensively demonstrate the performance of ISLRWR in DTIs prediction, we chose deepDTnet, MEDTI, NeoDTI, SGCL-DTI, DistMult, and ComplEx as baseline models to compare them with ISLRWR. We found that ISLRWR performs excellently on a variety of indicators.

To obtain direct auxiliary information on DTIs, we plotted the predictions of ISLRWR as a network connectivity map and found the more densely connected target common networks for interpretation. In addition, we provide some correctly predicted drug target pairs and new drug target pairs verified by the database as auxiliary information for drug discovery.

However, the present study had several limitations. Our methods only used direct association information from heterogeneous networks and did not use higher-order dependencies. For example, drugs and targets were associated with each other through multistep transit paths, and this type of information was referred to as metapaths by some scholars. In future research, we will consider further utilizing higher-order relations in higher-order network topological properties. In addition, although good performance was observed on the DTIs prediction task, generalizability could be lacking. Moreover, the findings of this study require further validation and generalization in other tasks or domains. In the future, we would like to apply our method of DTIs prediction to other association prediction tasks, such as lncRNA–miRNA interaction prediction [58, 59], metabolite–disease association prediction [60, 61] and numerous other tasks in biomedical forecasting [62–66]. These tasks have commonalities with the DTIs prediction task, and using graph network models for association prediction is currently a popular treatment.

## Conclusion

This paper aims to provide better performing and more usable network models for DTIs prediction than those currently available. The entirety of the model is enhanced by improving the individual parts of the model. In this paper, the MHRW algorithm, IMRWR algorithm, and proposed ISLRWR algorithm (as an improvement of the original RWR algorithm) are applied to achieve the overall performance improvement of the model. This approach proves to be helpful for the improvements of the model, however, the degree of innovation is limited. Therefore, in the future, researchers can develop completely new models or revolutionize the range of applications based on the current overall improvement of the model.
